# Transcriptome clarifies mechanisms of lesion genesis versus progression in models of Ccm3 cerebral cavernous malformations

**DOI:** 10.1186/s40478-019-0789-0

**Published:** 2019-08-19

**Authors:** Janne Koskimäki, Dongdong Zhang, Yan Li, Laleh Saadat, Thomas Moore, Rhonda Lightle, Sean P. Polster, Julián Carrión-Penagos, Seán B. Lyne, Hussein A. Zeineddine, Changbin Shi, Robert Shenkar, Sharbel Romanos, Kenneth Avner, Abhinav Srinath, Le Shen, Matthew R. Detter, Daniel Snellings, Ying Cao, Miguel A. Lopez-Ramirez, Gregory Fonseca, Alan T. Tang, Pieter Faber, Jorge Andrade, Mark Ginsberg, Mark L. Kahn, Douglas A. Marchuk, Romuald Girard, Issam A. Awad

**Affiliations:** 1 Neurovascular Surgery Program, Section of Neurosurgery, The University of Chicago Medicine and Biological Sciences, Chicago, IL USA; 2 Center for Research Informatics, The University of Chicago, Chicago, IL USA; 3 The Molecular Genetics and Microbiology Department, Duke University Medical Center, Durham, NC USA; 4 Department of Medicine, University of California San Diego, La Jolla, CA USA; 5 Department of Cellular and Molecular Medicine, University of California, San Diego, La Jolla, CA USA; 6 Department of Medicine and Cardiovascular Institute, University of Pennsylvania, Philadelphia, PA USA; 7 University of Chicago Genomics Facility, The University of Chicago, Chicago, IL USA

**Keywords:** Cerebral cavernous malformation, Cavernous angioma, Hemangioma, Ccm3, Mouse model, Transcriptome, miRNA

## Abstract

Cerebral cavernous malformations (CCMs) are dilated capillaries causing epilepsy and stroke. Inheritance of a heterozygous mutation in *CCM3/PDCD10* is responsible for the most aggressive familial form of the disease. Here we studied the differences and commonalities between the transcriptomes of microdissected lesional neurovascular units (NVUs) from acute and chronic in vivo *Ccm3/Pdcd10*^*ECKO*^ mice, and cultured brain microvascular endothelial cells (BMECs) *Ccm3/Pdcd10*^*ECKO*^.

We identified 2409 differentially expressed genes (DEGs) in acute and 2962 in chronic in vivo NVUs compared to microdissected brain capillaries, as well as 121 in in vitro BMECs with and without *Ccm3/Pdcd10* loss (fold change ≥ |2.0|; *p* < 0.05, false discovery rate corrected). A functional clustered dendrogram generated using the Euclidean distance showed that the DEGs identified only in acute in vivo NVUs were clustered in cellular proliferation gene ontology functions. The DEGs only identified in chronic in vivo NVUs were clustered in inflammation and immune response, permeability, and adhesion functions. In addition, 1225 DEGs were only identified in the in vivo NVUs but not in vitro BMECs, and these clustered within neuronal and glial functions. One miRNA *mmu-miR-3472a* was differentially expressed (FC = − 5.98; *p* = 0.07, FDR corrected) in the serum of *Ccm3/Pdcd10*^*+/−*^ when compared to wild type mice, and this was functionally related as a putative target to *Cand2* (cullin associated and neddylation dissociated 2), a DEG in acute and chronic lesional NVUs and in vitro BMECs. Our results suggest that the acute model is characterized by cell proliferation, while the chronic model showed inflammatory, adhesion and permeability processes. In addition, we highlight the importance of extra-endothelial structures in CCM disease, and potential role of circulating miRNAs as biomarkers of disease, interacting with DEGs. The extensive DEGs library of each model will serve as a validation tool for potential mechanistic, biomarker, and therapeutic targets.

**Electronic supplementary material:**

The online version of this article (10.1186/s40478-019-0789-0) contains supplementary material, which is available to authorized users.

## Introduction

Cerebral cavernous malformations (CCMs) also known as cavernous angiomas or hemangiomas, are clusters of leaky capillaries lined by endothelium presenting with aberrant angio-architecture. CCMs predispose 0.5% of the American population to a lifetime risk of hemorrhagic stroke and epilepsy [1, 2, 4]. The sporadic form of the disease shows a solitary lesion often connected to a developmental venous anomaly, while the familial form harbors multiple lesions throughout the brain and has been associated to an autosomal dominant mutation in one of the three CCM genes (*CCM1/KRIT1*, *CCM2/OSM* or *CCM3/PDCD10*) [8, 39, 62].

Although the exact pathway mechanisms of CCM disease are unclear, previous reports demonstrate that CCM genes regulate Rho-associated protein kinase (ROCK) activity [70]. A loss of one of CCM genes has been associated with increased ROCK activity resulting in dysregulated endothelial cell (EC) junctions and vascular hyperpermeability. These dysregulated biological processes result in a complex pathobiologic milieu involving an interplay of inflammation, angiogenesis, endothelial stress response, and the loss of endothelial barrier function processes [13, 28, 32–34, 37, 67, 73, 74, 76]. In the recent years, a number of biochemical in vitro and in vivo studies on engineered animal models of CCM disease have helped to unravel molecular mechanisms underlying the dysregulation of the CCM genes [28, 32, 33, 67, 76]. Numerous pathways have been reported in the genesis and maturation of CCM lesions, including the MEKK3-KLF2/4 [76], TGF-β/BMP [37], Wnt/β-catenin [17] and Notch pathways [72]. Despite progress in characterizing of the genetic basis of this disease, the pathobiological mechanism remains incompletely elucidated.

The acute murine models that have been engineered to study signaling aberrations driving CCM lesion genesis, harbor high lesion burden within the cerebellum [27, 70, 73]. However, these acute models only survive for a short period of time [73]. Therefore, typical chronic phenotypic characteristics of human lesions are missing such as iron deposits indicative of chronic hemorrhage, and inflammatory cell infiltration [73]. The chronic murine models more closely recapitulate the human disease including the stochastic development of new lesions throughout the brain over time, defective endothelial cell-cell junctions, iron deposition, and immune cell infiltration [73]. Finally, in vitro brain microvascular endothelial cells (BMECs) give opportunity to focus on the endothelial mechanisms associated to CCM disease [32, 33].

We hypothesized that phenotypical differences observed between acute and chronic models may be reflected in the respective expression of genes regulating cellular proliferation versus immune response and chronic bleeding. In addition, the majority of CCM studies have been focused on the ECs to elucidate CCMs genesis and maturation [37, 59, 74, 76]. However the contribution of the microenvironment around the lesional endothelium remains unclear [28, 34]. Indeed, CCM proteins are also expressed in other components of neurovascular units (NVUs) such as neurons, astrocytes or pericytes [34]. For this reason, we also assume that the phenotypic differences between in vivo and in vitro models may identify the contributions of other components of NVUs than ECs. Finally, we aimed to explore circulating miRNAs from *Ccm3* model that may identify new novel transcriptomic regulators and target genes. This is the first study comparing acute and chronic murine lesion NVU transcriptomes and DEGs with in vitro BMEC gene loss, and the first exploration of the emerging field of miRNA profiling in murine models of CCM.

## Material and methods

### Microdissected lesional NVUs from *Pdgfb*^*iCreERT2*^*Ccm3/Pdcd10*^*ECKO*^ acute model and corresponding controls

Six acute endothelial-specific *Pdgfb*^*iCreERT2*^*Pdcd10*^*fl/fl*^ mice were generated by breeding *Pdgfb*^*iCreERT2*^ and *loxP*-flanked *Ccm3/Pdcd10* exon 4 mice. At 1, 2 and 3 postnatal days (P1, P2 and P3), mice were administered 50 μg of tamoxifen intraperitoneally (Sigma Aldrich, St. Louis, MO) to induce Cre activity and endothelial *Ccm3/Pdcd10* gene loss (*Pdgfb*^*iCreERT2*^*Ccm3/Pdcd10*^*ECKO*^). Mice were anesthetized and sacrificed by decapitation at P9. Control non-lesional NVUs were collected from forebrain. Mouse brains were surgically removed and dropped into a 10% neutral buffered formalin fixative solution (Sigma Aldrich). Harvested brains were embedded in paraffin tissue blocks of 1 mm thickness. All sections were examined at × 1.5 magnification for quality and presence of CCM. Seven-μm thick tissue sections were mounted on Leica glass slides (Leica Biosystems Inc., Buffalo Grove, Illinois, USA) and stained with Paradise (Applied Biosystems, California, USA) according to the manufacturers’ protocols. The endothelial layer, including adjacent luminal and extra-luminal elements (i.e. NVUs) from CCMs and normal brain capillaries were then collected using laser capture microdissection and stored at − 80 °C. The NVUs of this model are referred to as “acute *in vivo* NVUs”.

### Microdissected lesional NVUs from *Pdgfb*^*iCreERT2*^*Ccm3/Pdcd10*^*ECKO*^ chronic model and corresponding controls

Five endothelial-specific *Pdgfb*^*iCreERT2*^*Pdcd10*^*fl/fl*^ mice were generated similarly as described above. Induction was performed with intraperitoneal injection of 50 μg at P4 and 5. Five littermates without tamoxifen injections were used as control. Mice were anesthetized and decapitated at P36–41. The mouse brains were then surgically extracted and harvested as described in the previous section. The NVUs of this model are referred to as “chronic *in vivo* NVUs”.

### BMECs of *Pdgfb*^*iCreERT2*^*Ccm3/Pdcd10*^ECKO^ and corresponding controls

The primary BMECs were isolated from three endothelial-specific conditional *Ccm3/Pdcd10* null mice (*Ccm3/Pdcd10*^*ECKO*^) generated using a *Pdgfb* promoter driven tamoxifen-inducible Cre recombinase, *Pdgfb*^*iCreERT2*^*; Pdcd10*^*fl/fl*^ [28, 32]. Following purification, BMECs were treated using 5 μM 4-hydroxytamoxifen for 48 h to induce allelic loss in ECs harboring the Cre-recombinase floxed allelic system. Next, the medium was replaced with medium lacking 4-hydroxytamoxifen, and BMECs were harvested after 168 h. Three control mice were similarly treated with 5 μM 4-hydroxytamoxifen. This model is referred to as “*in vitro* BMECs”.

### Circulating miRNome from *Ccm3/Pdcd10*^+/−^ mouse model and corresponding controls

Blood from 12 to 14 week-old littermate wild type and *Ccm3/Pdcd10*^*+/−*^ heterozygous (Pdcd10tm1.1Wami, MGI 5002631) mice on C57BL/6 background were collected by mandibular vein puncture. After collection of blood, serum was separated by using Z-gel tubes (Sarstedt, Nümbrecht, Germany) through centrifugation. Aliquots of 100 μl serum was collected and stored at − 80 °C until extraction of the circulating miRNAs.

### mRNAs and circulating miRNome extraction and cDNA library generation

RNAs from the acute and chronic in vivo NVUs were extracted using an RNA isolation kit (RNeasy® Micro Kit, Qiagen, Hilden, Germany). The cDNA libraries were generated using commercially low-input strand specific RNA-Seq kits (Clontech, California, USA), and sequenced on the Illumina HiSeq4000 platform using single-end 50 basepair (bp) reads. For in vitro BMECs, RNA was extracted using the TRIzol protocol (ThermoFisher, Waltman, MA). TruSeq Stranded mRNA Sample Prep Kit (Illumina, San Diego, CA) was used for library generation. RNA samples were sequenced using one yield single-end 75 bp reads. The cDNA library generation for in vitro BMECs is previously described [28, 32].

The circulating miRNA extraction from three *Ccm3/Pdcd10*^*+/−*^ and the three wild types was performed using the miRNeasy Serum/Plasma Kit (Qiagen, Hilden, Germany). The cDNA libraries were generated with commercially available Illumina small RNA-Seq kits (Illumina, San Diego, California, USA), and sequenced with the Illumina HiSeq4000 platform using single-end 50 bp reads.

Raw sequencing quality was assessed using FastQC (v0.11.8, www.bioinformatics.babraham.ac.uk/projects/fastqc/). The small RNA adapter sequences were trimmed from small RNA sequencing data with cutadapt 1.18 (https://cutadapt.readthedocs.io). The adapter trimmed reads were then mapped and quantified to the human mature miRNA database (miRBase 22) in a library mapping mode with sRNAbench standalone version [52], wrapped bowtie alignment (with alignment type = −n, seed length for alignment = 20, allowed number of mismatch = 0, minimum read count = 2 and maximum number of multiple mappings = 20). Low expressed miRNAs were then removed for further downstream analyses. Finally, the miRNA expression values were normalized following the trimmed mean of m-values (TMM) normalization methods with library size correction [50].

The differentially expressed miRNAs were identified between the *Ccm3/Pdcd10*^*+/−*^ genotype and wild type mice using R bioconductor package edgeR [38, 49]. The miRNA putative target genes were predicted with miRDB (http://mirdb.org/) [71]. The exploration and integrative analyses of miRNA-mRNA gene regulations were assisted using R [12].

### Statistical analyses and source data

**Fig. 1:**
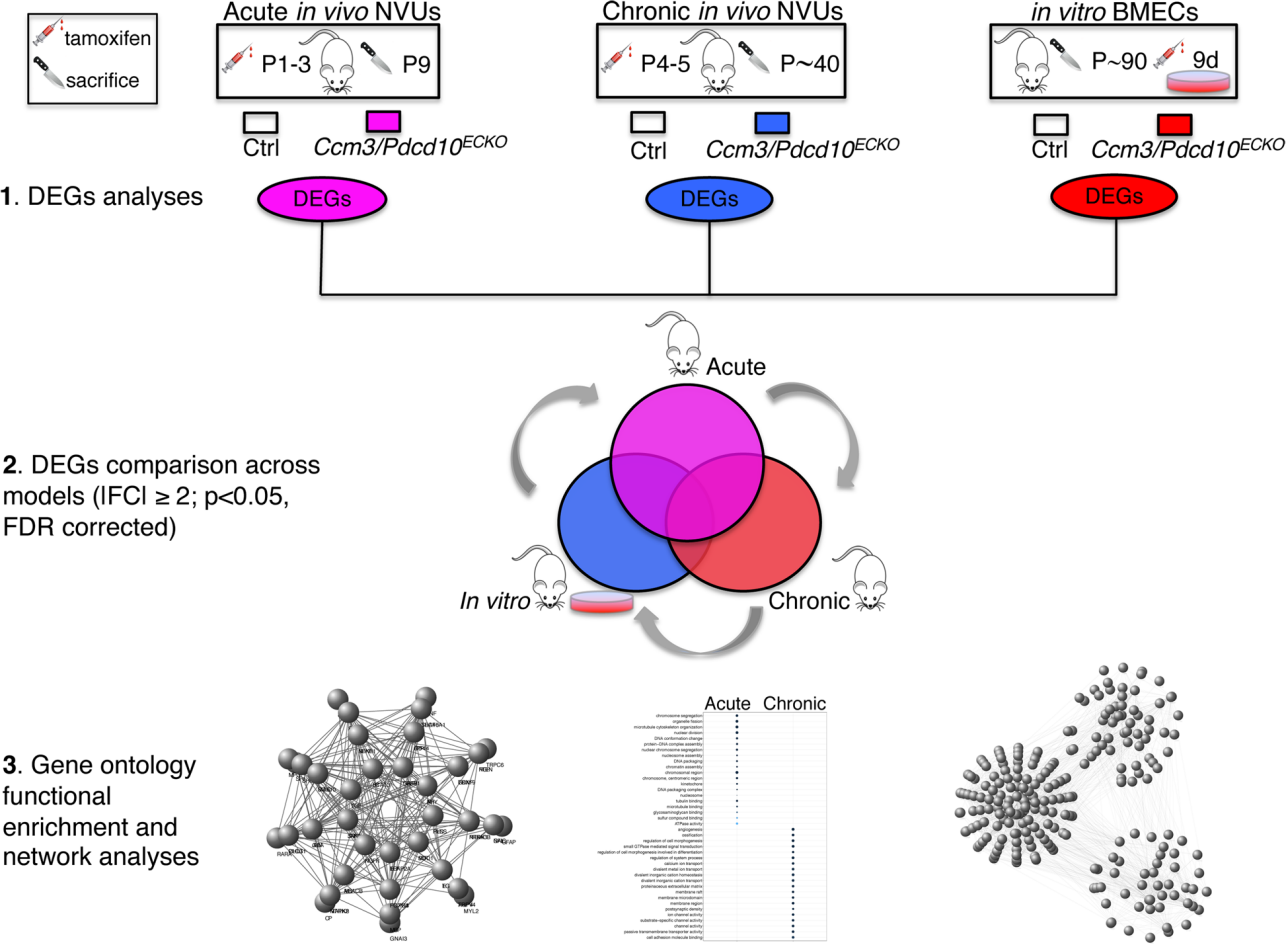
Flow diagram of the study. Three engineered pre-clinical models of CCM disease were cross-compared to identify the differences and commonalities between acute in vivo NVUs and chronic in vivo NVUs, as well as in vivo and in vitro models. We also studied the genes that are likely responsible for extra-endothelial pathological changes in CCM disease. The differentially expressed genes (DEGs) analysis was performed using DEseq2 for all models (fold change |FC| ≥ 2; *p* < 0.05, false discovery rate (FDR) corrected). DEGs of each model were compared with their respective healthy controls (|FC| ≥ 2; *p* < 0.05, FDR corrected). Lastly, we performed comprehensive gene ontology enrichment and network analyses

Work flow is presented in Fig. 1. Bioinformatics analyses (Additional file 1: Figure S1) are described in Additional file 1. Full lists of differentially expressed genes (DEGs) (fold change |FC| ≥ 2.0; *p* < 0.05 false discovery rate (FDR) corrected) as well as gene ontology (GO) lists are presented in Additional file 1. The raw sequencing data for the NVUs from the acute in vivo and chronic in vivo models used in this study are freely available in the National Center for Biotechnology Information Gene Expression Omnibus (GEO) database and are accessible through GEO series accession number GSE134005 and GSE134007. Data for in vitro BMEC model is accessible through GEO series accession number GSE123968. The raw miRNA sequencing data for *Ccm3/Pdcd10*^*+/−*^ mice is accessible through GEO series accession number GSE134006.

## Results

### DEGs and GO functions in acute in vivo NVUs, chronic in vivo NVUs, and in vitro BMECs

**Fig. 2:**
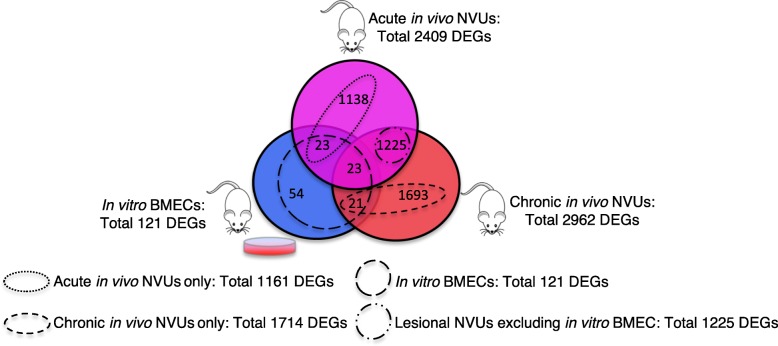
Venn diagram of the different comparisons performed. We studied the differences observed between the 1161 differentially expressed genes (DEGs) only identified in the acute in vivo neurovascular units (NVUs) and the 1714 DEGs only identified in the chronic in vivo NVUs. In addition the 121 DEGs identified in the in vitro BMECs’ were compared to the 1225 DEGs common between acute and chronic in vivo NVUs, but were not present in the in vitro BMECs. (For all: fold change |FC| ≥ 2; *p* < 0.05, false discovery rate corrected)

The analyses identified 2409 DEGs in acute in vivo NVUs (fold change |FC| ≥ 2.0; *p* < 0.05, FDR corrected) compared to the respective controls (Fig. 2, Additional file 1: Figure S2, Additional file 2: Table S1). The top 10 |FC| (by absolute FC) DEGs in the acute in vivo model included *Barhl1*, *Gabra6*, *Lbx1*, *Pax2*, *En2*, *Fat2*, *Pcp2*, *Pax3*, *Cnpy1 and Fsip2*. The top 20 |FC| DEGs contributed to neurodevelopmental related GO functions such as brain and spinal cord development as well as neuronal differentiation (*p* < 0.05, FDR corrected) (Additional file 1: Figure S3, Additional file 3: Table S2 and Additional file 4: Table S3).

In addition, 2962 DEGs were identified in chronic in vivo NVUs (fold change |FC| ≥ 2.0; *p* < 0.05, FDR corrected) compared to the respective controls (Fig. 2, Additional file 1: Figure S2, Additional file 2: Table S1). The top 10 |FC| DEGs included genes *Gabra6*, *Fat2*, *Ddn*, *Gpr88*, *Cbln3*, *Foxg1*, *Kcnv1*, *Pcp2*, *Ngp*, and *Cnpy1*. The top 20 |FC| DEGs contributed to neurodevelopmental related GO functions as well, but also included regulation of cytokine secretion, immune response and axonogenesis (*p* < 0.05, FDR corrected) (Additional file 1: Figure S3, Additional file 3: Table S2 and Additional file 4: Table S3).

Furthermore, 121 DEGs were identified in in vitro BMECs (fold change |FC| ≥ 2.0; *p* < 0.05, FDR corrected) compared to the respective controls (Fig. 2, Additional file 1: Figure S2, Additional file 2: Table S1). The top 10 |FC| DEGs included *Aplnr*, *Slc38a5*, *Eln, Ptprr*, *Ltbp2*, *Mgp*, *Sncg*, *Tgfbi*, *St14*, *and F2rl2*. The top 20 |FC| DEGs contributed to GO functions related to extracellular matrix, heparin binding, and regulation of G-protein coupled signaling (*p* < 0.05, FDR corrected) (Additional file 1: Figure S3, Additional file 3: Table S2 and Additional file 4: Table S3).

### DEGs, enriched GO functions and gene networks identified only in acute in vivo NVUs and only in chronic in vivo NVUs

**Fig. 3:**
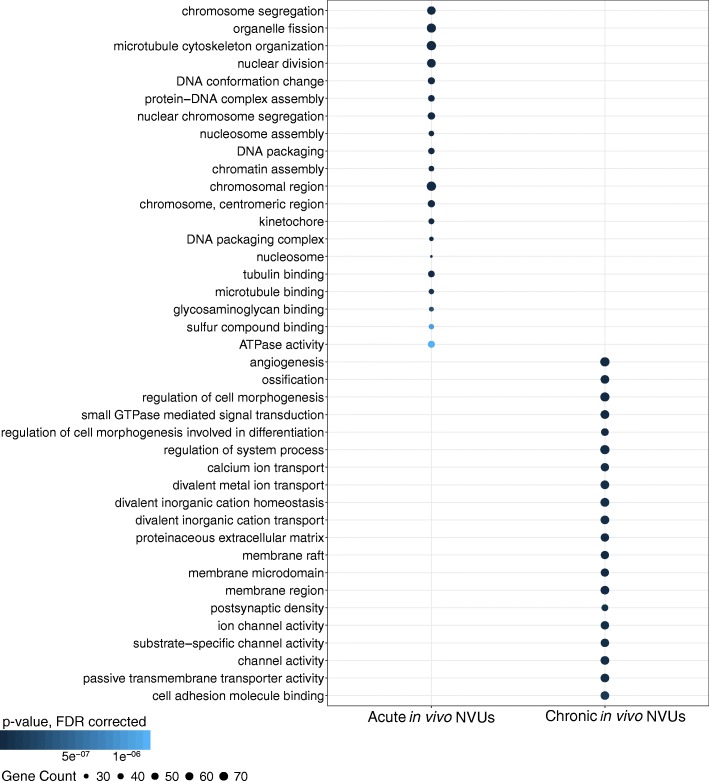
Gene ontology (GO) functional enrichment analysis of differentially expressed genes (DEGs) only identified in the acute in vivo neurovascular units (NVUs) and only in the chronic in vivo NVUs. The GO analysis of the DEGs only identified in the acute in vivo NVUs found 375 enriched GO terms, while 702 enriched GO terms were found with the DEGs only identified in the chronic in vivo NVUs (both: *p* < 0.01, false discovery rate corrected)

**Fig. 4:**
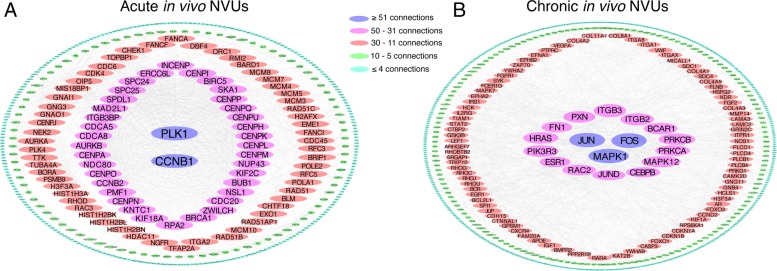
Functional interaction networks of the differentially expressed genes (DEGs) only identified either the acute or the chronic in vivo neurovascular units (NVUs). **a** The functional interaction network analysis using the DEGs only identified in the acute in vivo NVUs showed that *PLK1* and *CCNB1* are highly connected (with more than 50 edges) and 36 others DEGs with 31–50 edges. **b** The network analysis using the DEGs only identified in chronic in vivo NVUs showed that *JUN, FOS, MAPK1* were highly connected (with more than 50 edges), and 14 others DEGs with 31–50 edges. The human homologous genes corresponding to the DEGs identified in each mouse models were used for the network analysis

The analyses identified 1161 genes that were only dysregulated in acute in vivo NVUs (|FC| ≥ 2.0; *p* < 0.05, FDR corrected) that represented DEGs specific for acute model studied (Fig. 2 and Additional file 5: Table S4). The functional GO enrichment analysis showed 375 enriched GO terms (*p* < 0.01, FDR corrected) (Additional file 6: Table S5). The top 20 GO terms were related to chromosomal, microtubular, and DNA functional modulation (Fig. 3). The network analysis showed that 36 DEGs were highly connected (more than 30 edges) (Fig. 4a). Polo-like kinase 1 (*PLK1*) and cyclin B1 (*CCNB1)* showed more than 50 edges.

Similarly, 1714 genes were only dysregulated in chronic in vivo NVUs (|FC| ≥ 2.0; *p* < 0.05, FDR corrected) that represented DEGs specific for chronic model studied (Fig. 2 and Additional file 5: Table S4) and were enriched in 702 GO terms (*p* < 0.01, FDR corrected) (Additional file 6: Table S5). The top 20 GO terms were related to functions of angiogenesis, ion transportation, GTPase transduction, and cell adhesion (Fig. 3). Among these 1714 DEGs, 14 DEGs were highly connected (more than 30 edges), three of which showed more than 50 edges including *JUN, FOS* and *MAPK1* (Fig. 4b).

**Fig. 5:**
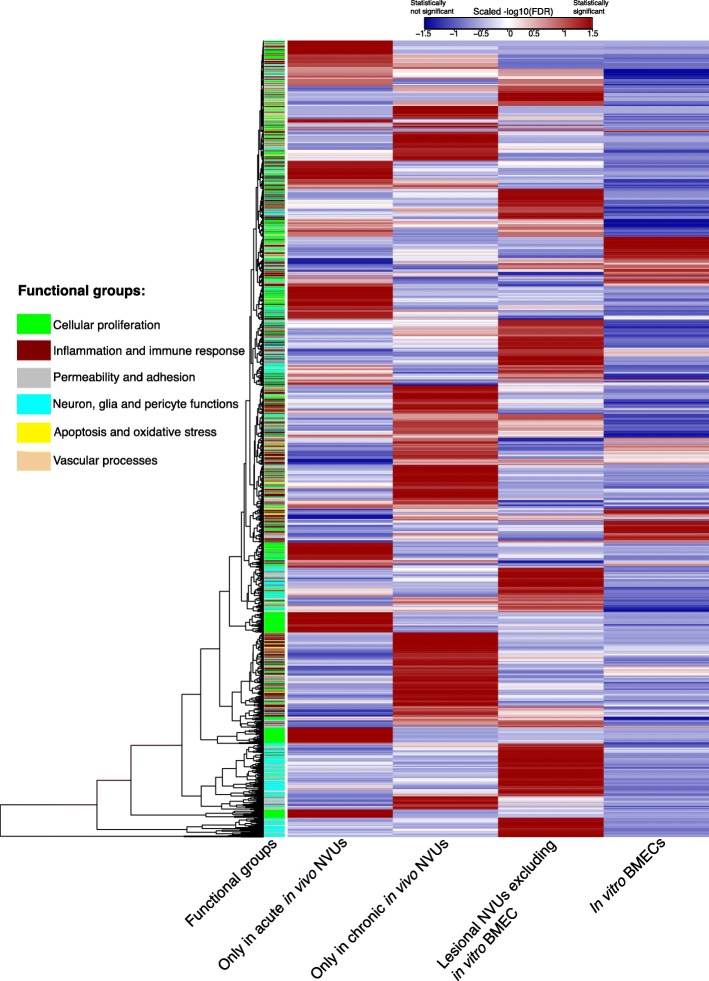
Heatmap of functional differences between the acute and chronic lesional in vivo neurovascular units (NVUs), and in vitro brain microvascular endothelial cells (BMECs). Gene ontology terms were queried into six functional groups based on a systematic literature review of CCM disease. Functional groups included cellular proliferation (green), inflammation and immune response (maroon), permeability and adhesion (grey), neuron, glia and pericyte functions (light blue), apoptosis and oxidative stress (yellow), and vascular processes (light brown). Five clusters including cellular proliferation processes were identified in the acute in vivo NVUs. In the chronic in vivo NVUs, four clusters were related to inflammation, immune response, permeability, and adhesion functions. In the lesional NVUs excluding in vitro BMECs, three clusters were identified including neural, glial, and pericyte functions clusters. In the in vitro BMECs, two functional clusters were observed and did not included specific functions. Clustering of the groups were based on their Euclidean distance. The statistical significance in the heatmap was calculated and presented based on the -log10 false discovery rate (FDR) corrected *p*-values (Red significant, blue not significant)

To compare the GO functions across models we defined six functional categories associated with CCM disease, including inflammation and immune response, extra-endothelial cell environment (neuron, glia and pericyte functions), cell proliferation, apoptosis and oxidative stress, vascular processes, permeability, and adhesion (Additional file 1). The functional clustered dendrogram generated using the Euclidean distance highlighted the most important functional differences between acute and chronic in vivo NVUs (Fig. 5, Additional file 7: Table S6). The DEGs identified from the acute in vivo NVUs were clustered in cellular proliferation GO functions. In the chronic in vivo NVUs, the DEGs were clustered into inflammation and immune response, permeability, and adhesion functions (Fig. 5).

### DEGs, enriched GO functions and gene networks identified only in in vivo lesional NVUs and in vitro BMECs

**Fig. 6:**
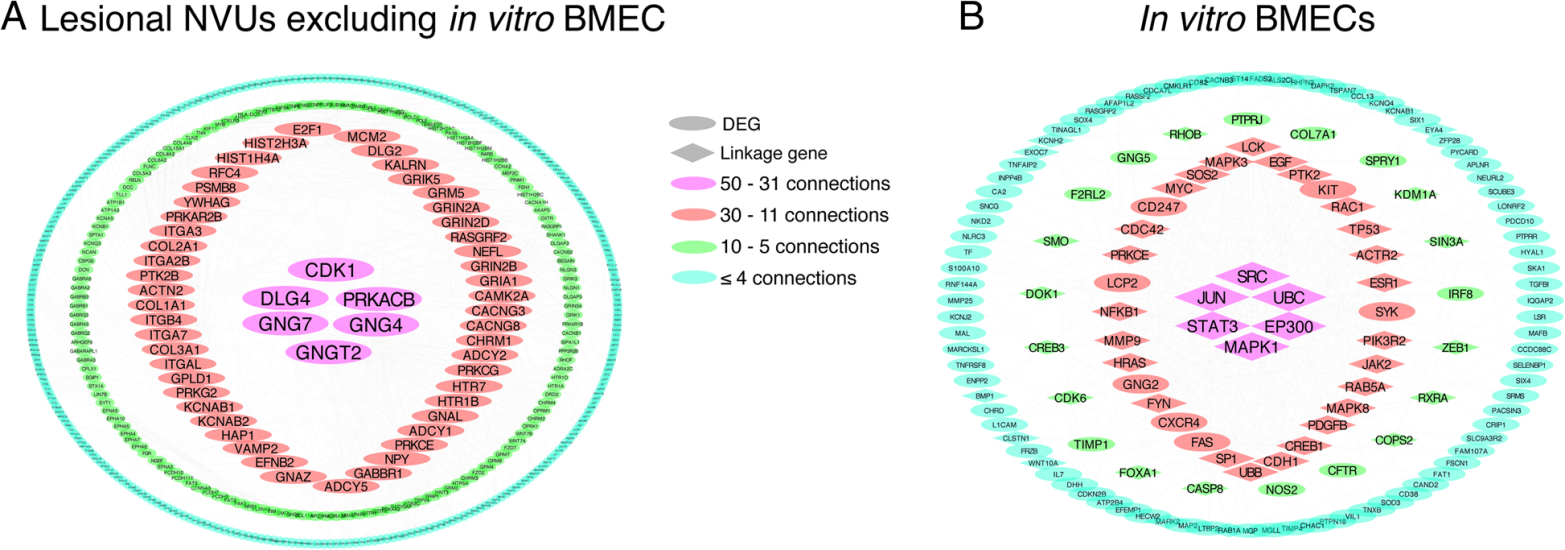
Functional interaction networks of differentially expressed genes (DEGs) in lesional neurovascular units excluding in vitro brain microvascular endothelial cell (BMEC) and in vitro (BMECs). **a** The functional interaction network analysis of the 1225 DEGs (fold change |FC| ≥ 2; *p* < 0.05, false discovery rate (FDR) corrected) identified as common between in vivo and in vitro and not in the in vitro BMECs, showed six highly connected genes (with more than 50 edges) including *CDK1*, *DLG4*, *GNG4*, *GNG7*, *PRKACB* and *GNGT2*, and 50 DEGs with 31–50 edges. **b** The functional interaction network analysis with linkage genes of the 121 DEGs (|FC| ≥ 2; *p* < 0.05, FDR corrected) identified in the in vitro BMECs showed 6 DEGs with 11–30 edges including *KIT*, *SYK*, *FAS*, *CXCR4*, *GNG2*, *LCP2,* and *CD247*. Furthermore, nine DEGs were identified with 5 to 10 edges: *PTPRJ*, *COL7A*, *SPRY*, *IRF8*, *CFTR*, *NOS2*, *TIMP1*, *F2RL2* and *GNG5*. Genes with 31–50 edges were linkage genes and included *JUN*, *STAT3*, *MAPK1*, *EP300*, *UBC* and *SRC*. The human homologous genes corresponding to the DEGs identified in each mouse models were used for the network analysis

We then studied genes that are likely responsible for extra-endothelial pathological changes in CCM disease by excluding the DEGs identified only in the in vitro BMECs from the pool of commonly differentially expressed genes between acute and chronic in vivo NVU models (Fig. 2). We identified 1225 DEGs common between in vivo acute and chronic NVU models that were not observed in the in vitro BMECs (|FC| ≥ 2.0; *p* < 0.05, FDR corrected) (Fig. 2 and Additional file 5: Table S4). This comparison excluded DEGs originating from cultured endothelial cell population, leaving 1225 extra-endothelial DEGs for further study. Functional GO enrichment analyses showed 877 GO terms (*p* < 0.01, FDR corrected) (Additional file 8: Table S7). The top 20 enriched GO terms were related to axonogenesis, synaptic transmission, ion channel activity, and regulation of neurotransmitters. Network analysis identified 6 genes (*CDK1*, *DLG4*, *GNG4*, *GNG7*, *PRKACB* and *GNGT2*) highly connected (with more than 30 edges) (Fig. 6a).

**Fig. 7:**
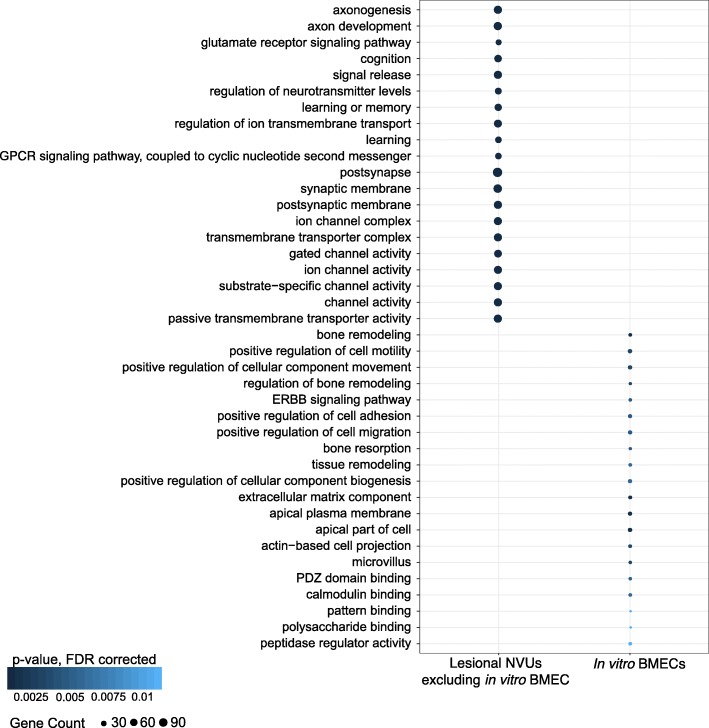
Gene ontology (GO) functional enrichment analyses of differentially expressed genes (DEGs) only observed in the lesional neurovascular units excluding in vitro brain microvascular endothelial cell (BMEC) and in vitro BMECs. The GO enrichment analysis of the DEGs only identified in the in vivo lesional neurovascular units identified 877 GO terms (*p* < 0.01, false discovery rate (FDR) corrected). In addition, the GO enrichment analysis of the DEGs only identified in in vitro BMECs found 226 GO terms (*p* < 0.05, FDR corrected)

The functional GO enrichment analysis of the 121 DEGs (|FC| ≥ 2.0; *p* < 0.05, FDR corrected) identified in the in vitro BMECs showed 226 GO terms (*p* < 0.05, FDR corrected) (Additional file 8: Table S7). The top enriched GO functions were related to cell motility, cell migration, ERBB signaling, and cell polarization (Fig. 7). The most connected DEGs (more than 10 edges) were *KIT*, *SYK*, *FAS*, *CXCR4*, *GNG2*, *LCP2* and *CD247* (Fig. 6b). In addition, *JUN*, *STAT3*, *MAPK1*, *EP300*, *UBC* and *SRC* were linkage genes with more than 31 edges.

The functional clustered dendrogram showed that the GO functions in lesional NVUs excluding in vitro BMEC were enriched in three clusters including neuronal, neuroglial and pericytic functions (Fig. 5, Additional file 7: Table S6). In the in vitro BMECs, two clusters were identified and included non-specific functions (Fig. 5, Additional file 7: Table S6).

### Common DEGs across three models

**Fig. 8:**
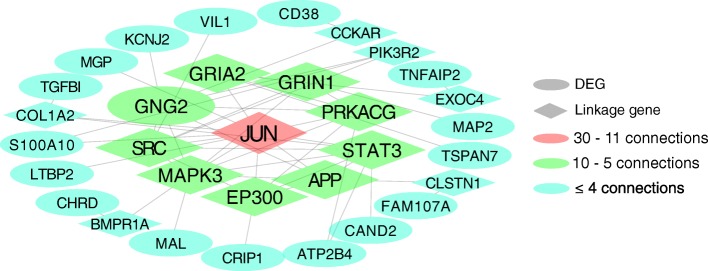
Functional interaction network of 23 differentially expressed genes (DEGs) common between all three models. The network analysis identified that the most connected linkage gene was *JUN* with 11 edges. In addition, *GNG2* was most connected DEG with 6 connections, other DEGs showed less than five connections. The human homologous genes corresponding to the DEGs identified in each mouse models were used for the network analysis

Twenty-three DEGs were common across all three mouse models (|FC| ≥ 2.0; *p* < 0.05, FDR corrected) (Fig. 2 and Additional file 5: Table S4). The network analysis showed *GNG2* as the most connected DEG (Fig. 8). In addition, *JUN* was identified as the most connected linkage gene with 11 edges. We further queried the different transcriptomes of each model and observed that *JUN* (FC = 2.01; *p* < 0.001, FDR corrected) was only dysregulated in the chronic in vivo NVUs (Additional file 5: Table S4).

### Circulating miRNome in *Ccm3/Pdcd10*^*+/−*^ mouse model and putative targets

One miRNA *mmu-miR-3472a* was differentially expressed (FC = − 5.98; *p* = 0.07, FDR corrected) in the serum of *Ccm3/Pdcd10*^*+/−*^ when compared to wild type mice (Additional file 1: Supplemental Results, Figure S4, Additional file 9: Table S8). The integration analyses of *mmu-miR-3472a* within the 23 DEGs common between the three models identified *Cand2* (cullin associated and neddylation dissociated 2) as a putative target. Further analyses showed that *Cand2* was down-regulated in in vitro BMECs (FC = − 2.03; *p* < 0.001, FDR corrected), and up-regulated in the acute and chronic in vivo NVUs (FC = 2.20; and FC = 2.27 respectively; *p* < 0.001, FDR corrected).

## Discussion

Our group recently published a cross-comparison analysis across multiple species and genotypes including DEGs in human CCM lesions and with the loss of CCM genes in *Caenorhabditis elegans* and in vitro BMEC cultures [28]. Analysis of transcriptomes from the murine lesions, comparing stages of lesion development, have never been accomplished previously, nor any exploration of circulating miRNAs in the same mice. Here we cross-compared the transcriptome profiles of three models of the most aggressive familial form of CCM disease, including acute and chronic in vivo models, as well as in vitro BMECs. We aimed to identify DEGs characterizing acute and chronic components of CCM disease. We also identified the genes that are likely responsible for extra-endothelial pathological changes in CCM disease. Finally, we also studied the circulating miRNome from serum of *Ccm3/Pdcd10* mice.

### The most differentially expressed genes in in vivo and in vitro models show different functions

In the in vivo models, the functional enrichment analyses of top-20 DEGs were related to the central nervous system and neuronal development, while in the in vitro model they were related to extracellular matrix. In addition, chronic model acquired enriched cytokine secretion functions, not present in the acute model.

In the acute in vivo NVUs model, four genes including *Lbx1*, *Pax2*, *Pax3*, *Neurog1* were identified among the top-20 DEGs, and were related to neural development [30, 42, 43]. *Barhl1* also belonged to the top-20 DEGs and genetically engineered *Barhl1*^*−/−*^ mice model develop medulloblastoma-phenotype [46]. Currently, these top DEGs are not established in CCM disease.

However, among the top-20 DEGs, there were a number of DEGs identified in the transcriptome of chronic in vivo NVUs related to inflammation. We identified *Crtam*, which determines the CD4^+^ cytotoxic T-lymphocyte lineage [66], *Ngp*, which functions as a cytokine expressed from CD4^+^ helper T-cells, and *Mmp12*, which is an important regulator of leukocyte trafficking, cytokine secretion and T-helper cell differentiation [41]. Our previous study has shown that B-cell depletion reduces the maturation of CCM lesion in the mouse model [63]. However, there are no studies reporting T-cell functions or T-B-cell interactions in CCM disease.

The top-20 DEGs identified in the in vitro BMEC model included transforming growth factor β induced (*Tgfbi*) and latent Tgfβ binding protein (*Ltbp2*), which have both been linked to cancer invasion and metastasis [22, 68, 69, 77]. This finding is interesting since the importance of Bmp6 and Tgfβ signaling for endothelial-to-mesenchymal transformation in CCM disease has been established [37]. Although pathological processes are different in cancer and CCM, this purports the role of similar yet novel *Tgfbi* and *Ltbp2* in CCM disease. In addition, *Ltbp2* has also been shown to be dysregulated in infantile hemangiomas, linking it to other vascular pathologies [65]. Similarly, *Tnxb* was highly upregulated, which encodes tenascin X, which has been shown to promote epithelial-mesenchymal transformation by activating latent Tgfβ [3]. The role of *Tnxb* has not been studied yet in CCM disease. Further studies may address the role of this gene and its protein product during endothelial-mesenchymal transformation in CCM pathology.

### Transcriptomic and functional differences between acute and chronic in vivo NVUs

Acute models have been widely used to study signaling and molecular aberrations in CCM disease [13, 73, 76]. Our results showed that the enriched GOs were mostly related to cell division processes. This finding suggests that during lesion genesis, ECs are actively dividing, which supports the phenotypical observation of a high and rapidly developed lesion burden [73]. EC proliferation may be driven by functions related to chromosome segregation, nucleosome assembly, DNA packaging, kinetochore, chromatin assembly, nuclear division and microtubule binding. The network analyses identified two genes with more than 50 connections, polo-like kinase 1 (*PLK1*) and cyclin B (*CCNB1*), which are both important cell cycle regulators [5, 24]. Cyclin B following phosphorylation by Cdk1, which is also up-regulated in our transcriptome of acute in vivo NVUs, forces cells into mitosis [24]. On the other hand, ubiquitin-dependent degradation of cyclin B instead drives cells into mitotic exit [18]. Unfortunately, changes in phosphorylation or ubiquitination status are post-transcriptional modifications and cannot be observed in transcriptomic data. Plk1 is the most important co-operator for cyclin B-Cdk1 to continue cell division, despite the importance of oscillations and positive feedback loop of cyclin B-Cdk1 [5, 24]. Plk1 together with phosphorylated cyclin B-Cdk1 promotes centrosome maturation and regulates the kinetochore’s proper attachment to the mitotic spindle [5, 24]. Interestingly, *Cdc25* is also up-regulated in our transcriptome of acute in vivo NVUs, and is responsible for phosphorylation and proper localization of cyclin B-Cdk1 and Plk1 [7].

In addition, unphosphorylated retinoblastoma protein (*Rb1* gene), that is related to cellular proliferation, restricts the cell cycle through binding E2f1–3 transcription factors [6]. Interestingly, *E2f1* and *E2f2* genes were up-regulated in our transcriptome data. These two genes are also transcription factors promoting expression of *Cyclin A* and *Cdc6* [6], which are cell cycle progression genes, and were up-regulated in our acute model. Furthermore, we observed an upregulation of *Cnne2*, which encodes for the Cyclin E2 protein responsible for hyperphosphorylation of Rb protein [14, 64]. The conformational changes of Rb, due to hyperphosphorylation, prevents the binding of E2f1–3 transcription factors, and thus promote cell cycle progression [14]. In our dataset *Rb1* was not differentially expressed. However, the hyperphosphorylation of the Rb due to an increased expression of Cyclin E may explain why cells pass the utmost important G1/S checkpoint. In addition, an up-regulation of Cyclin B and *Cdk1* is the main mechanism for passing the next G2/M check point, which are necessary for continuing beyond this all or none check point [24, 47]. The dysregulated genes identified only in the transcriptome of acute in vivo NVUs transcriptome may explain the pathological processes occurring during lesion genesis. However, the initial mechanism initiating the cellular growth and division remain unclear. These results ultimately suggest that defective cell cycle regulation may be one of the main contributors to the development of CCM lesions.

In contrast, current phenotypic evidence suggest the importance of immune and inflammation processes in CCM lesion maturation after an initial lesion has formed [60, 73]. Our network analysis of DEGs only observed in the chronic in vivo NVUs identified three highly connected genes (with more than 50 edges) including *JUN, FOS* and *MAPK1. Jun, Fos* and *Jund* are subunits that compose the AP-1 transcription factor [56], which is important for regulating cytokine production, immune, and stress responses [48, 75]. Experimental evidence showed that the loss of function of CCM proteins increases intracellular reactive oxygen species (ROS) and oxidative stress [48]. This pathological process may contribute to the upregulation of inflammatory transcription factors including NF-κB, AP-1 and PPAR-γ, leading to the production and release of cytokines and chemokines [48]. Interestingly, dysfunctional vascular remodeling and increased permeability has also been linked to a dysregulation of c-Jun [15, 35, 51]. Furthermore, c-Jun has already been shown to be up-reregulated after *Krit1* loss [19]. This loss of *Krit1* is also associated with a significant increase in intracellular ROS levels, while normal Krit1facilitates low ROS levels resulting in downregulation of cyclin D1 expression leading to a cell transition from proliferative growth to quiescence [19]. Finally, dysfunctional vascular remodeling and increased permeability has also been linked to a dysregulation of c-Jun.

*Mapk1* and *Mapk12* belong to the MAPK superfamily that may play a role in MEKK3-KLF2/4 pathway during CCM genesis [76]. Our result are consistent with previous findings that have shown communication between MEKK3-KLF and RAS/MAPK1 pathways [9, 67, 76]. *Ccm1*, *Ccm2* and *Ccm3* form an adaptor complex interacting with MAPK pathways, mediating angiogenesis and apoptosis in CCM lesion [9, 67, 76]. We used the *Ccm3* model, rather than the *Ccm1* and *Ccm2* models, suggesting that different *CCM* mutations may eventually activate similar biological processes and signaling pathways that eventually trigger lesion formation. Loss of *kri-1* in *C. elegans* has also been reported to cause non-autonomous effects on the MAPK pathway inducing apoptosis [9]. Furthermore, it is important to note that the role of the MEKK3-KLF2/4 pathway in lesion maturation has not been yet clearly studied. It has been previously suggested that MAPK pathways contribute to inflammation and immune responses [40]. Probably, the most well-known contributor in this process is NF-κB [40]. Our results showed that *Mapk1* is downregulated while stress reactive *Mapk12* is upregulated [21]. We also observed an up-regulation of *Rps6ka1*, which encodes ribosomal S6 kinase (p90^rsk^, MAPK-activated protein kinase 1). P90^rsk^ regulates transcription factors NF-κB, c-Fos, and CREB modulating local inflammation responses [40, 44]. This suggests that MAPK pathways may also be involved in inflammation response after lesion is in a more mature state.

c-Jun and c-Fos together with stress induced MAPKs and p90^rsk^ may be interesting candidates that bridge lesion genesis and maturation. Indeed, the network analysis with linkage genes showed that the 23 DEGs common between the three models were highly connected with *JUN*. Furthermore, *JUN* was only dysregulated in the chronic in vivo model and not in the acute in vivo model. This result suggests that *JUN* may have a role during the development of specific features associated with the chronic state of the disease such as inflammation and maturation processes, relevant to clinically active lesions. Finally, paxillin (from *PXN gene*) and integrin β subunit 2 (from *ITGB2 gene*) are important proteins that increase leukocyte-endothelial interactions and promote leukocyte transmigration through vascular ECs [10, 45]. These two genes maybe important regulators of leukocyte migration into the lesion site which is also an observed feature in the chronic models [73].

### The comparison of lesional NVUs excluding in vitro BMEC DEGs and in vitro BMECs address the importance of whole NVU in CCM lesion pathobiology

Finally, we identified the genes that are likely responsible for extra-endothelial pathological changes in CCM disease. The relative contribution of each cellular type in the transcriptome of the in vivo NVUs models cannot be determined in this study. However, the overall importance of extra-endothelial processes and functions occurring around in vivo lesions can be discussed. The GOs enriched in lesional NVUs excluding in vitro BMEC DEGs indicated clear clustering of cellular functions related to neuronal or glial functions as hypothesized. Lesion genesis/maturation may cause reactive changes in gene expression in the other components of the NVUs including neurons, astrocytes or pericytes. Although the effects of CCM gene dysregulation on other cell types are not fully elucidated, it has been showed that neural specific conditional mouse *Ccm3* mutants develop dilated capillaries and CCM-like lesions [34]. This cell non-autonomous phenomenon supports the idea that CCM lesions can develop as a result of non-EC mutation [34]. Our current results comparing transcriptomes of NVUs and single cell population of extracted BMECs suggest that mutation occurring specifically only in the EC may cause major gene expression changes in the extra-endothelial environment, secondary to the primary mutation and gene expression changes in the ECs. However, this observation needs to be further validated and addressed with single cell studies. The network analysis showed that *Gng4, Gng7, Gngt2* were highly connected, which all encode γ subunits of heterotrimeric G proteins. These proteins are related to signal transduction and postsynaptic signaling, together with postsynaptic density protein 95 (PSD95) encoded by the gene *Dlg4* [26, 57, 58]. Notably, mice with deficiency in G protein γ subunits have dramatically increased susceptibility to seizures [58], which is a common manifestation of certain CCM lesions in patients [2]. Furthermore, it has been widely shown that PSD95 controls excitation balance in the synapses [26]. These results address the importance of extra-endothelial cells in pathological cascades that may be related to the CCM disease phenotype.

In the in vitro BMEC model, the enriched GO functions that mainly describe current knowledge of the pathogenesis of CCMs, including Mapk signaling, angiogenesis, actin filament organization, cell migration, cell-matrix adhesion and phosphoinositide signaling [16, 23, 28, 55, 73, 76]. This suggests that in vitro BMECs are a reliable model to study basic pathological endothelial mechanisms involved in CCM disease since many of the pathological mechanism identified are already established in in vivo CCM models [28, 32, 33, 55, 73, 74, 76]. However, the BMEC model completely lacks the extra-endothelial effects that may restrict translating results to in vivo. Understanding this limitation, these observations importantly confirm the translational reliability of the widely used mouse BMEC CCM model and encourage testing new novel mechanistic hypotheses also in vitro.

### Circulating miRNA targeted one common gene across all models

Circulating miRNome have been a major interest to the research community during recent decades and rigorous scientific effort have led major medical breakthroughs in the oligonucleotide research field [31]. To our knowledge, only one study has sequenced the intra-lesional miRNome of resected human CCM lesions [25]. Our group has also previously identified circulating miRNAs in human patients [36]. Here we identified *mmu-miR-3472a* that was differentially expressed in the serum of *Ccm3/Pdcd10*^*+/−*^ mice. Importantly, we found that *mmu-miR-3472a* has the putative target *Cand2* that was commonly dysregulated in the transcriptomes of all three models. *Cand2* is closely linked to the signaling cascades of cullins [11, 20]. Cullins are important in protein ubiquitination process and thus affect protein degradation through the proteasome [20]. Cand protein interacts with cullins and regulates activity of Skp, Cullin, and F-box containing complex (SCF complex), which is an E3 ubiquitin ligase complex [20]. This pathway regulates the activity of ubiquitin dependent protein degradation in proteasome [20]. Interestingly, cullins have also been associated with endothelial barrier function through mediating vascular endothelial-cadherin expression [53], and also regulating *VEGF2R* expression in vascular ECs [54]. In addition, the Cullin-3-Rbx1-KCTD10 complex regulates endothelial barrier function through ubiquitination of RhoB [29]. This suggests that *Cand2* may play a role in CCM disease. However, these novel observations and associations await further mechanistic study.

### Limitations

In this study, we only used engineered *Ccm3* mice models. The results may not apply to *Ccm1* or *Ccm2* disease models [28]; however, the lesions of two different genotypes are histologically indistinguishable. In addition, the *Ccm3* murine models have a more robust lesion burden. Furthermore, the mature multicavernous lesions show all known phenotypic signatures of the human lesion, including ECs lining the caverns lacking the respective CCM protein, defective EC barrier, high EC proliferative index, chronic bleeding with iron deposition, and a robust inflammatory response [61, 73].

We also used rigorous *p*-value with FDR correction (*p* < 0.05) and FC threshold (|FC | ≥ 2.0), which guaranteed very reliable statistical results. However, a high |FC| threshold may leave some DEGs outside of the analyses that may have a role in pathogenesis of CCM disease. In addition, we compared three biological models to each other, in order to classify and categorize genes and their enriched functions. The comparison and categorization give us the opportunity to understand more closely specific gene expression patterns and functions. This approach can be further developed by using single cell technology that may allow for separation of in vivo ECs completely from the other components of NVUs.

The circulating miRNome was determined from mice *Ccm3/Pdcd10*^*+/−*^ and then compared to a wild type. *Ccm3/Pdcd10*^*+/−*^ mice develop a low lesion burden and multicavernous lesions are rarely observed [59]. However, the *Ccm3/Pdcd10*^*+/−*^ model is genotypically similar to the familiar human *CCM3* disease, having a heterozygous mutation in the *CCM3* gene in every cell. In addition, this first exploratory miRNA study included only three heterozygous mice and three wild type mice. However, this data suggests that at least one circulating miRNA is specific to *Ccm3/Pdcd10*. Furthermore, we plan to perform miRNA studies in others preclinical models of CCM disease in the future in order to understand their role more comprehensively and find new targets to be studied in humans.

While our study did not provide mechanistical validation for the results, this study described the transcriptomic differences in the models investigated and provides strong candidates for further validation in CCM disease.

## Conclusions

Our results show transcriptomic and functional differences between acute and chronic as well as between in vivo and in vitro models. The acute model was characterized by cell proliferation functions, whereas the chronic model showed functions of inflammation. Our results also highlight the importance of extra-endothelial structures during genesis and maturation of CCM lesion. The libraries of the three models generated within this study will provide validation tool for potential mechanistic, biomarker, and therapeutic targets. They will also generate hypotheses to be tested mechanistically in the CCM research field.

## Additional files

Additional file 1: Supplemental Methods. Supplemental Results. Supplementary References. **Figure S1.** Statistical flow chart. **Figure S2.** Volcano plots of differentially expressed genes (DEGs) in acute and chronic in vivo neurovascular units (NVUs) and in vitro brain microvascular endothelial cells (BMECs). **Figure S3.** Dot plot comparing enriched gene ontology functions (*p* < 0.05, false discovery rate corrected) of top 20 genes by absolute fold change of acute in vivo neurovascular units (NVUs), chronic in vivo NVUs, and in vitro brain microvascular endothelial cells. **Figure S4.** Functional interaction network of the putative targets of *mmu-miR-3472a* within the differentially expressed genes commonly identified between acute and chronic in vivo lesional neurovascular units. (PDF 707 kb)

Additional file 2: **Table S1.** List of identified differentially expressed genes in each of three models (fold change |FC| ≥ 2, *p* < 0.05, false discovery rate corrected). (XLSX 643 kb)

Additional file 3: **Table S2.** Top 20 genes by fold change for acute in vivo neurovascular units (NVUs), chronic in vivo NVUs and in vitro brain microvascular endothelial cells models (fold change |FC| ≥ 2; *p* < 0.05, false discovery rate corrected). (XLSX 18 kb)

Additional file 4: **Table S3.** List of enriched gene ontology functions for top 20 genes by fold change in each model (*p* < 0.05, false discovery rate corrected). (XLSX 14 kb)

Additional file 5: **Table S4.** List of unique differentially expressed genes for all models according to the Venn diagram of the models (fold change |FC| ≥ 2.0; *p* < 0.05, false discovery rate corrected). (XLSX 745 kb)

Additional file 6: **Table S5.** List of gene ontology terms for acute in vivo neurovascular units (NVUs) only and chronic in vivo NVUs only (*p* < 0.01, false discovery rate corrected). (XLSX 160 kb)

Additional file 7: **Table S6.** 1876 gene ontology (GO) terms used for generating the heatmap. (XLSX 157 kb)

Additional file 8: **Table S7.** List of gene ontology terms for lesional neurovascular units excluding in vitro brain microvascular endothelial cell (BMEC) differentially expressed genes (*p* < 0.01, false discovery rate (FDR) corrected) and in vitro BMECs (*p* < 0.05, FDR corrected). (XLSX 145 kb)

Additional file 9: **Table S8.** List of 105 putative targets of *mmu-miR-3472a* within the differentially expressed genes common between acute and chronic in vivo neurovascular units. (XLSX 34 kb)

## Data Availability

The raw sequencing data for the NVUs from the acute in vivo and chronic in vivo models used in this study are freely available in the National Center for Biotechnology Information Gene Expression Omnibus (GEO) database and are accessible through GEO series accession number GSE134005 and GSE134007. Data for in vitro BMEC model is accessible through GEO series accession number GSE123968. The raw miRNA sequencing data for *Ccm3/Pdcd10*^*+/−*^ mice is accessible through GEO series accession number GSE134006.

## Abbreviations

BMEC: Brain microvascular endothelial cell
CCM: Cerebral cavernous malformation
DEG: Differentially expressed gene
FC: Fold change
FDR: False discovery rate
GEO: Gene Expression Omnibus
GO: Gene ontology
KRIT1: Krev interaction trapped 1
NVU: Neurovascular unit
OSM: Osmosensing scaffold for MEKK3
PDCD10: Programmed cell death 10
PDGFB: Platelet Derived Growth Factor Subunit B
ROCK: Rho-associated protein kinase
TMM: Trimmed mean of m-values
