# Supplementary material for: Transcriptome clarifies mechanisms of lesion genesis versus progression in models of Ccm3 cerebral cavernous malformations
Source: Acta Neuropathol Commun. 2019 Aug 19;7:132. doi: 10.1186/s40478-019-0789-0 (PMC6699077; doi:10.1186/s40478-019-0789-0)
Supplement: Supplementary file 1 — Supplemental Methods. Supplemental Results. Supplementary References. Figure S1. Statistical flow chart. Figure S2. Volcano plots of differentially expressed genes (DEGs) in acute and chronic in vivo neurovascular units (NVUs) and in vitro brain microvascular endothelial cells (BMECs). Figure S3. Dot plot comparing enriched gene ontology functions (p < 0.05, false discovery rate corrected) of top 20 genes by absolute fold change of acute in vivo neurovascular units (NVUs), chronic in vivo NVUs, and in vitro brain microvascular endothelial cells. Figure S4. Functional interaction network of the putative targets of mmu-miR-3472a within the differentially expressed genes commonly identified between acute and chronic in vivo lesional neurovascular units. (PDF 707 kb) [file 40478_2019_789_MOESM1_ESM.pdf]

Supplemental Material

Transcriptome Clarifies Mechanisms of Lesion Genesis Versus Progression in Models of Ccm3 Cerebral Cavernous Malformations

Janne Koskimäki[*,1]; Dongdong Zhang[*,1]; Yan Li[2]; Laleh Saadat[1]; Thomas Moore[1]; Rhonda Lightle[1]; Sean P. Polster[1]; Julián Carrión-Penagos[1]; Seán B. Lyne[1]; Hussein A. Zeineddine[1]; Changbin Shi[1]; Robert Shenkar[1]; Sharbel Romanos[1]; Kenneth Avner[1]; Abhinav Srinath[1]; Le Shen[1]; Matthew R. Detter[3]; Daniel Snellings[3]; Ying Cao[1]; Miguel A. Lopez-Ramirez[4]; Gregory Fonseca[5]; Alan T. Tang[6]; Pieter Faber[7]; Jorge Andrade[2]; Mark Ginsberg[4]; Mark L. Kahn[6]; Douglas A. Marchuk[3]; Romuald Girard[1**]; Issam A. Awad[1**]

From:

[1]Neurovascular Surgery Program, Section of Neurosurgery, The University of Chicago Medicine and Biological Sciences, Chicago, IL

[2]Center for Research Informatics, The University of Chicago, Chicago, IL

[3]The Molecular Genetics and Microbiology Department, Duke University Medical Center, Durham, NC

[4]Department of Medicine, University of California San Diego, La Jolla, CA

[5]Department of Cellular and Molecular Medicine, University of California, San Diego, La Jolla, CA

[6]Department of Medicine and Cardiovascular Institute, University of Pennsylvania, Philadelphia, PA

[7]University of Chicago Genomics Facility, The University of Chicago, Chicago, IL

*Equal contribution

** Equal contribution as corresponding author

Correspondence to:

Issam A. Awad, MD, MSc, FACS, MA (hon), Section of Neurosurgery, University of Chicago Medicine, 5841 S. Maryland, MC3026/Neurosurgery J341, Chicago, IL 60637.

Telephone +1 773-702-2123; Fax +1 773-702-3518. E-mail iawad@uchicago.edu

Supplemental Methods

Bioinformatics analyses

The quality of raw sequencing reads was assessed by FastQC (v0.11.2; http://www.bioinformatics.babraham.ac.uk/projects/fastqc/). The post-alignment quality control was evaluated using RSeQC[1], and Picard tools (v1.117; http://broadinstitute.github.io/picard/). Reads of each models were mapped on the mouse genome reference (GRCm38)[2]. Gene transcripts were assembled and quantified using featureCounts with respect to corresponding reference genome gene annotations[3]. The differentially expressed genes (DEGs) for each model were identified using DESeq2[4], with a correction for batch effect if necessary. For each model, DEGs were identified in respective their appropriate control described in the main manuscript, **Materials and Methods** section. The genes were considered differently expressed if they showed an absolute fold change (FC) equal or greater than 2 ($|FC| \geq 2$) with a $p < 0.05$ with an adjusted false discovery rate (FDR) corrected.

We defined the pools of DEGs only observed in each models to study the difference between the acute and chronic *in vivo* neurovascular units (NVUs), and then between *in vivo* and *in vitro* models (Fig. 2). The DEGs only identified in the chronic lesional *in vivo* NVUs were differentially expressed (DE) in the chronic lesional NVUs and not in acute *in vivo* lesional NVUs. Using similar approach, the DEGs only identified in the acute *in vivo* lesional NVUs were DE only in the acute *in vivo* lesional NVUs and not in chronic *in vivo* lesional NVUs. We then studied genes that are likely responsible for extra-endothelial pathological changes in cerebral cavernous malformation (CCM) disease by excluding the DEGs identified only in the *in vitro* brain microvascular endothelial cells (BMECs) from the pool of commonly DE between *in vivo* acute and chronic NVU models.

Gene ontology (GO) enrichment analyses were conducted with R bioconductor package clusterProfiler. The heatmaps were constructed based on the -log10 false discovery rate (FDR) corrected p-value of GO terms related to CCM disease with row scaling implemented in R using heatmap.2 function. A complete linkage clustering method was used to explore the similarity of those selected GO terms based on their Euclidean distance. The terms used to query the GO database[5] (www.geneontology.org) to build the heatmaps were selected based on the words commonly used in CCM research. We first performed a systematic electronic review on PubMed

database of peer-reviewed articles published between February 15, 2008 and February 15, 2018, using the following key terms: (cerebral cavernous malformation [Title/Abstract] OR cerebral vascular malformation [Title/Abstract] OR cerebrovascular malformation [Title/Abstract] OR CCM1 [Title/Abstract] OR CCM2 [Title/Abstract] OR CCM3 [Title/Abstract] OR Krit1 [Title/Abstract] OR PDCD10 [Title/Abstract] OR MGC4607 [Title/Abstract] OR brain permeability [Title/Abstract]) AND (english [Language]) NOT (case report [Publication Type] NOT liver [Title/Abstract] NOT surgery [Title/Abstract] NOT management [Title/Abstract]) NOT treatment [Title/Abstract]).

The frequency of each word was then assessed within the 474 abstracts found on PubMed using R software. Finally, these words were used to generate six categories for CCM related GO terms: cellular proliferation (green), inflammation and immune response (maroon), permeability and adhesion (grey), neuron, glia and pericyte functions (light blue), apoptosis and oxidative stress (yellow), and vascular processes (light brown).

The network analyses were performed with ReactomeFIViz[6] in Cytoscape (http://www.cytoscape.org/) based on Reactome functional interaction network analysis platform (version 2016)[7]. The human homologous genes corresponding to the DEGs identified in each mouse models were used for the network analysis. The human homologous genes were annotated based on the National Center for Biotechnology Information HomoloGene database. Out of 18,038 corresponding human genes in the database, 16,200 (around 90%) genes from mouse have corresponding human homologous genes. The entire functional interaction network was constructed by merging interactions extracted from human curated pathways and predicted interactions from machine learning approach[7]. We defined the highly connected the genes with at least 30 edges in the pool of DEGs only identified in *in vivo* acute and *in vitro* chronic models. However, due to a lower number of input DEGs for the *in vitro* BMEC transcriptome we considered genes with at least 10 edges as highly connected.

DE miRNAs were analyzed as described in the main manuscript **Materials and Methods** section. Network analyses of miRNA target genes were performed with ReactomeFIViz[6] in Cytoscape (http://www.cytoscape.org/) based on Reactome functional interaction network analysis platform (version 2016)[7], as described above.

Supplemental Results

RNA quality

The acute and chronic *in vivo* lesional *Ccm3/Pdcd10*^{ECKO} NVUs as well as the normal NVUs showed an average estimated RNA concentration of 2.2±2.8 ng/µl and an average RNA integrity number (RIN) of 2.3±0.3. For the BMEC model and the respective controls, the average estimated RNA concentration and RIN were 148.0±65.2 ng/µl and 7.9±0.5 respectively.

Circulating *mmu-miR-3472a* and putative targets in the transcriptome common between chronic and acute *in vivo* lesional NVUs

The integration analysis identified 105 putative targets of *mmu-miR-3472a* within the 1248 DEGs common between acute and chronic *in vivo* NVUs (**Fig. S4 and Table S8**). Utilizing the network analysis with linkage genes *mmu-miR-3472a* putatively targets *PRKACB* and *COL1A1*, which have more than 10 connections. In addition, *NFKB1*, *FOS*, *JUN* and *STAT1* had more than 10 connections and are inflammation related genes. *CAND2* was connected to *NFKB1*, *FOS*, *JUN* through *STAT1*.

Supplementary References

1 Wang L, Wang S, Li W. RSeQC: quality control of RNA-seq experiments. *Bioinformatics* 2012;28(16):2184-2185.

2 Dobin A, Davis CA, Schlesinger F*, et al.* STAR: ultrafast universal RNA-seq aligner. *Bioinformatics* 2013;29(1):15-21.

3 Liao Y, Smyth GK, Shi W. featureCounts: an efficient general purpose program for assigning sequence reads to genomic features. *Bioinformatics* 2014;30(7):923-930.

4 Love MI, Huber W, Anders S. Moderated estimation of fold change and dispersion for RNA-seq data with DESeq2. *Genome Biol* 2014;15(12):550.

5 Ashburner M, Ball CA, Blake JA*, et al.* Gene ontology: tool for the unification of biology. The Gene Ontology Consortium. *Nat Genet* 2000;25(1):25-29.

6 Wu G, Dawson E, Duong A*, et al.* ReactomeFIViz: a Cytoscape app for pathway and network-based data analysis. *F1000Res* 2014;3:146.

7 Wu G, Feng X, Stein L. A human functional protein interaction network and its application to cancer data analysis. *Genome Biol* 2010;11(5):R53.

Supplemental Figures and Tables

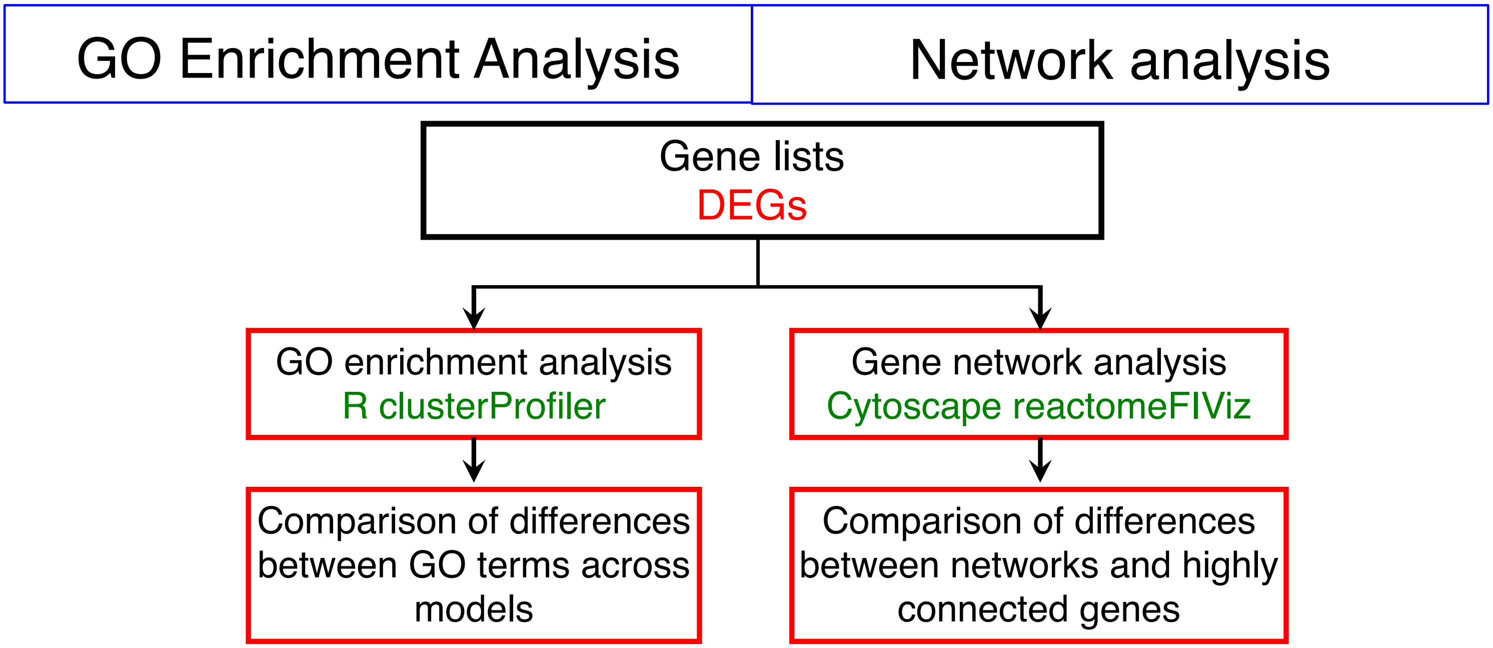

Fig. S1 Statistical flow chart. Approach 1 utilized comparison of gene ontology (GO) analyses and comparison of GO terms across models and specific differentially expressed gene (DEG) pools. GO enrichment analyses were performed using RclusterProfiler. In approach 2, respective groups were analyzed with ReactomeFIViz on Cytoscape 3.7.1. platform for functional gene interaction network analyses.

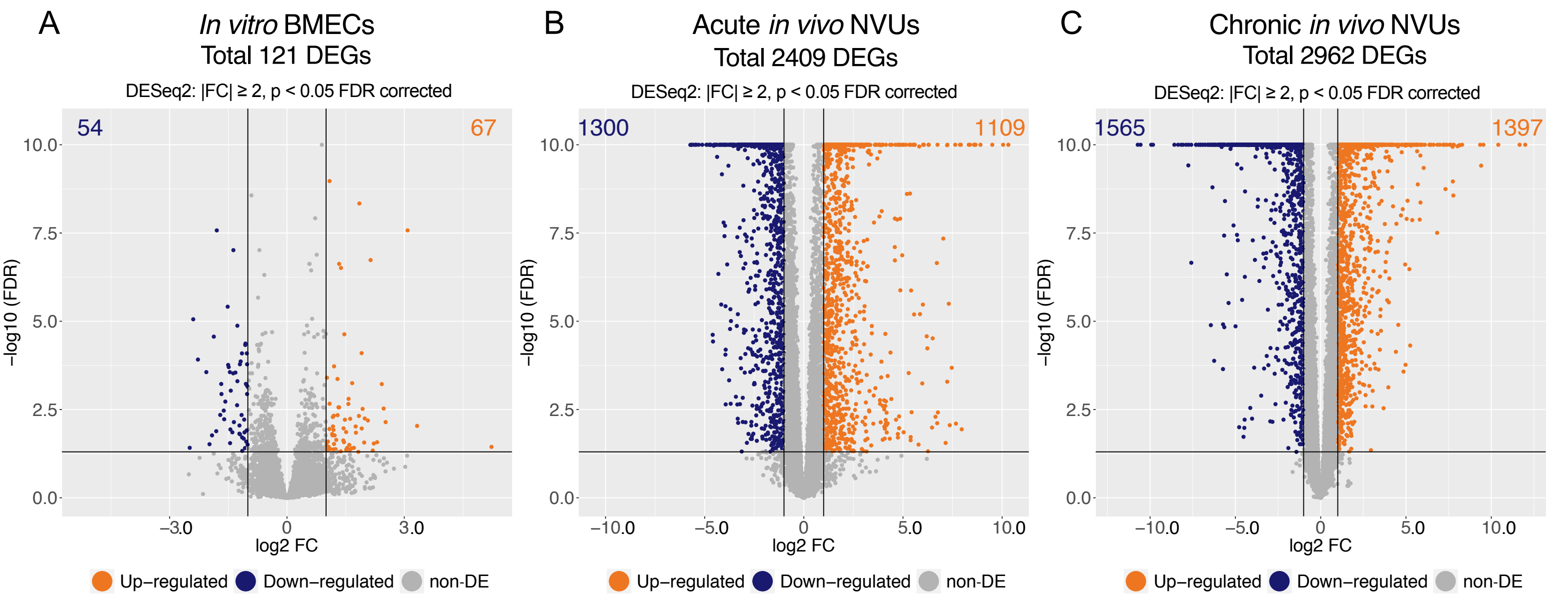

Fig. S2 Volcano plots of differentially expressed genes (DEGs) in acute and chronic *in vivo* neurovascular units (NVUs) and *in vitro* brain microvascular endothelial cells (BMECs) The transcriptome analyses identified (**A**) 121 DEGs in *in vitro* BMECs, (**B**) 2409 DEGs in acute *in vivo* NVUs, (**C**) 2962 DEGs in chronic *in vivo* NVUs. All DEGs were considered at a fold change $|FC| \geq 2$; $p < 0.05$, false discovery rate (FDR) corrected. Each dot represents an individual DEG with compared to the respective control. Red dots represent up-regulated DEGs and blue dots represents down-regulated DEGs. Results are presented as x-axis $\log_2$ FC and y-axis $\log_{10}$ FDR corrected p-value.

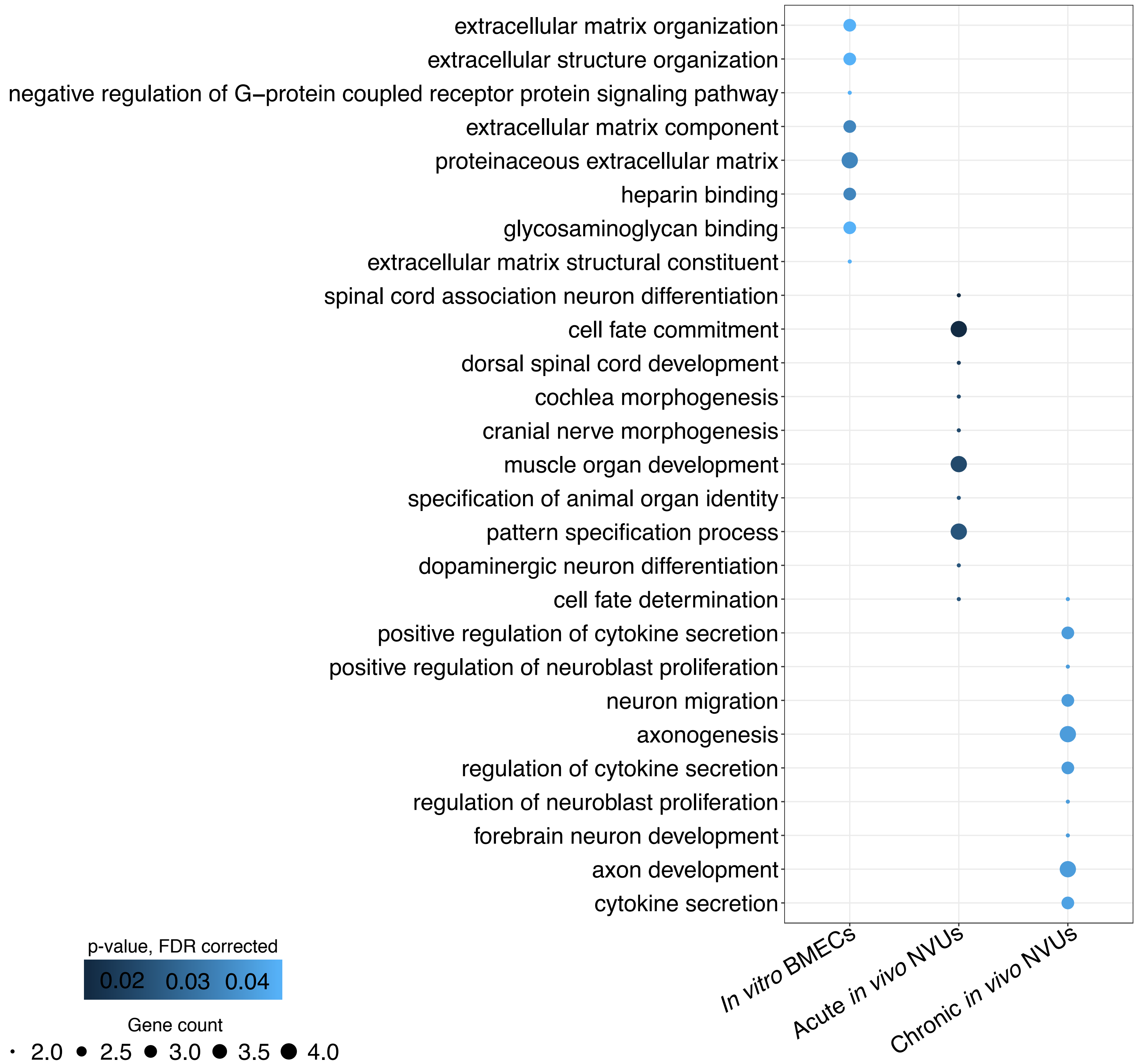

Fig. S3 Dot plot comparing enriched gene ontology functions ($p < 0.05$, false discovery rate corrected) of top 20 genes by absolute fold change of acute *in vivo* neurovascular units (NVUs), chronic *in vivo* NVUs, and *in vitro* brain microvascular endothelial cells.

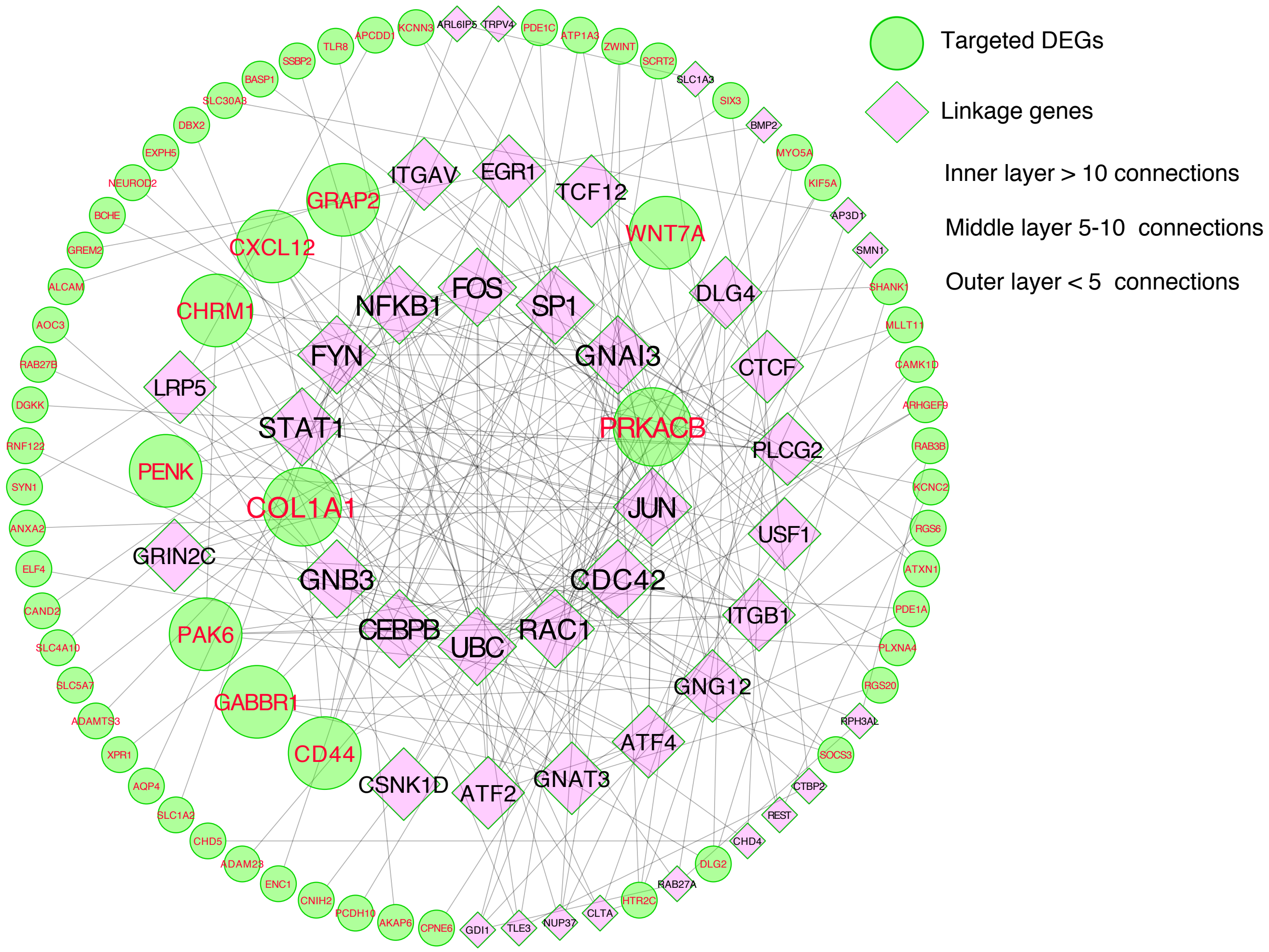

Fig. S4 Functional interaction network of the putative targets of *mmu-miR-3472a* within the differentially expressed genes commonly identified between acute and chronic *in vivo* lesional neurovascular units. The integration analysis identified a total of 105 putative target genes. Circulating miR-3472a targeted PRKACB and COL1A1 that have more than 10 connections. *NFKB1*, *FOS*, *JUN* and *STAT1* were identified as linkage genes with more than 10 connections. *CAND2* was connected to *NFKB1*, *FOS*, *JUN* through *STAT1*. The human homologous genes corresponding to the DEGs identified in each mouse models were used for the network analysis.

Table S1 List of identified differentially expressed genes in each of three models (fold change |FC| ≥ 2, $p < 0.05$, false discovery rate corrected).

Table S2 Top 20 genes by fold change for acute *in vivo* neurovascular units (NVUs), chronic *in vivo* NVUs and *in vitro* brain microvascular endothelial cells models (fold change |FC| ≥ 2; $p < 0.05$, false discovery rate corrected).

Table S3 List of enriched gene ontology functions for top 20 genes by fold change in each model ($p < 0.05$, false discovery rate corrected).

Table S4 List of unique differentially expressed genes for all models according to the Venn diagram of the models (fold change |FC| ≥ 2.0; $p < 0.05$, false discovery rate corrected).

Table S5 List of gene ontology terms for acute *in vivo* neurovascular units (NVUs) only and chronic *in vivo* NVUs only ($p < 0.01$, false discovery rate corrected).

Table S6 1876 gene ontology (GO) terms used for generating the heatmap.

Table S7 List of gene ontology terms for lesional neurovascular units excluding *in vitro* brain microvascular endothelial cell (BMEC) differentially expressed genes ($p < 0.01$, false discovery rate (FDR) corrected) and *in vitro* BMECs ($p < 0.05$, FDR corrected).

Table S8 List of 105 putative targets of *mmu-miR-3472a* within the differentially expressed genes common between acute and chronic *in vivo* neurovascular units.
